# Complex Extract of *Polygonatum sibiricum* and *Nelumbinis semen* Improves Menopause Symptoms via Regulation of Estrogen Receptor Beta in an Ovariectomized Rat Model

**DOI:** 10.3390/nu15112443

**Published:** 2023-05-24

**Authors:** Doori Park, Jee-Eun Yoon, Boram Choi, Yoon-Jae Lee, In-Hyuk Ha

**Affiliations:** Jaseng Spine and Joint Research Institute, Jaseng Medical Foundation, 538, Gangnam-daero, Gangnam-gu, Seoul 06110, Republic of Korea; pdr315@gmail.com (D.P.); jee1512@gmail.com (J.-E.Y.); bohosun@jaseng.org (B.C.); goodsmile8119@gmail.com (Y.-J.L.)

**Keywords:** menopause symptoms, menopause, estrogen receptor, *Polygonatum sibiricum*, *Nelumbinis semen*, herbal medicine

## Abstract

Menopause is a hormone-deficiency state that causes facial flushing, vaginal dryness, depression, anxiety, insomnia, obesity, osteoporosis, and cardiovascular disease as ovarian function decreases. Hormone-replacement therapy is mainly used to treat menopause; however, its long-term use is accompanied by side effects such as breast cancer and endometriosis. To identify the effect of a complex extract of *Polygonatum sibiricum* (*PS*) and *Nelumbinis semen* (*NS*) on improving menopause without side effects, an ovariectomized rat model was established to analyze several menopause symptoms. Compared to single extracts, the complex extract restored vaginal epithelial cell thickness and decreased serotonin concentration by increasing the estrogen receptors ERα (ESR1) and ERβ (ESR2), depending on the ratio. Although the complex extract exerted a lower weight-loss effect than the single extracts, improved blood-lipid metabolism was observed after increasing high-density lipoprotein cholesterol levels and decreasing low-density lipoprotein cholesterol and triglyceride levels, and ovariectomy-induced osteoporosis was alleviated by suppressing osteoclast production. Thus, by increasing only ERβ expression without regulating ERα expression in the uterus, the complex extract of PS and NS may be a natural treatment for improving menopause symptoms without side effects, such as endometriosis.

## 1. Introduction

Climacteric refers to when ovarian function declines owing to disease or aging, and menopause-related physical and psychological changes occur. It is a concept that encompasses both early and late menopause [1]. With the gradual increase in average life expectancy, women now spend approximately 1/3 to 1/2 of their lives in menopause, which is an emerging social problem [2]. As menopause progresses, women experience facial flushing, tachycardia, sweating, depression, anxiety, and insomnia due to acute deficiency of female hormones and suffer chronic diseases such as osteoporosis and cardiovascular disease due to chronic deficiency, which is known as climacteric syndrome [3,4]. Given that these hormonal changes reduce muscle mass in climacteric women, cause obesity by promoting abdominal fat accumulation, and increase the prevalence of metabolic syndrome, the need to provide treatment for climacteric women is gradually becoming more apparent [5].

Hormone replacement therapy (HRT), which involves direct administration of synthetic estrogen and progestin, is widely used to treat menopause symptoms; however, it causes weight gain, nausea, abdominal distension, and edema, and severe side effects such as breast cancer, endometrial cancer, and cardiovascular disease have been reported to accompany its long-term use [6,7]. Thus, natural bioactive compounds with fewer side effects are essential for research on alleviating menopause symptoms.

Estrogen (E2) is a hormone with the greatest decrease during the climacteric period. It is synthesized in the ovary or testis and adrenal gland [8], and its response signal is regulated through E2 receptors, ERα (ESR1) and ERβ (ESR2), which have distinct roles in addition to several typical physiological roles, such as ovarian development and function, and cardiovascular system protection. ERα plays a more prominent role in the mammary gland and uterus, and in maintaining skeletal homeostasis and regulating metabolism, whereas ERβ has a more profound impact on the central nervous and immune systems [9,10]. Cell hyperproliferation is generally regulated according to ERα expression level in tissues such as the breast and uterine tissues [10]. In the vaginal epithelium, E2 induces epithelial cell proliferation through ERα and ERβ to improve vaginal dryness due to menopause [11]. However, administering E2 in the uterus may cause endometriosis owing to difficulties in regulating endometrial cell hyperproliferation due to ERα expression [12,13]. A recent study showed that c-fos expression increases in the endometrium of patients with endometriosis and is associated with increased 17β-E2 levels in the serum [14]. Owing to the various side effects caused by the ERα activity of these synthetic E2s, there is a growing interest in administering selective ERβ-activators, which are considered safer than E2, as nonspecific ER activators. One of these activators, MF101, a herbal extract, has been shown to reduce the frequency of facial flushing in clinical trials [15,16]. 

*Polygonatum sibiricum* (PS) has been traditionally used in functional foods and oriental medicine in Korea and China [17,18], and has been reported to exhibit many therapeutic effects and biological activities such as antioxidative and antiaging properties, osteoporosis suppression, neuroprotection, immunity enhancement, diabetes improvement, fatigue improvement, and anticancer effects [19,20,21]. PS enhances blood-lipid metabolism and antioxidant activity in menopausal mice [22]. Moreover, “Jaseng JStrogen,” which has been clinically used to treat various menopause symptoms, contains PS. In a previous study, PS regulated the ERα/ERβ ratio through the progesterone receptor, alleviating menopause symptoms without endometrial cancer and hypertrophy [23]. 

*Nelumbinis semen* (NS) is a dried product obtained from the rind of ripe lotus seeds, a perennial aquatic plant belonging to the Nymphaceae family. In China and Korea, NS is traditionally used to promote the function of the spleen, kidney, heart, and eyes and prevent diarrhea and dysentery, and it is effective in cases of anxiety, sensitivity, or insomnia because of its calming effect [24]. According to recent studies, NS is rich in various bioactive compounds that improve human health, such as alkaloids and flavonoids [24,25], which are mainly used to treat neurological disorders, insomnia, and postmenstrual depression in women [26]. In addition, NS has antiviral, hepatoprotective, antioxidative, memory-improving, anti-inflammatory, and hair growth-promoting effects [27,28,29]. Moreover, “Jaseng Chunggong-Dan,” which is clinically used to treat chronic fatigue, lack of energy, memory loss, and lack of fluid, contains NS. 

In this study, it was hypothesized that the complex extract of PS and NS would exhibit greater efficacy in menopause improvement than a single extract, and its efficacy was evaluated using ovariectomized (OVX) rat models. The complex extract of PS and NS enhanced various menopause symptoms, such as blood lipid metabolism, vaginal dryness, osteoporosis, depression, and endometriosis, in postmenopausal OVX rat models. Furthermore, the inhibitory effect of the complex extract of PS and NS against potential side effects of endometriosis through selective regulation of ERβ in the endometrium was investigated.

## 2. Materials and Methods

### 2.1. Preparation of PS and NS Extracts

PS, NS, and *Leonurus japonicus* (LJ) were purchased from Green M. P. Pharm. Co. Ltd. (Gyeonggi-do, Republic of Korea). PS and NS were mixed in PS:NS ratios (*w*/*w*) of 1:1 and 2:1. The aqueous extracts of LJ, PS, and NS and the mixtures were prepared with distilled water and brought to a boil using a reflux device maintained at 88 °C for 6 h. After cooling to 20–22 °C, they were filtered using a filter paper, and the filtrates were, after cooling to −20 °C, freeze-dried using a freeze dryer (Ilshin BioBase Co., Ltd., Gyeonggi-do, Republic of Korea) to obtain dry PS, NS, and extract mixture, which were stored at −20 °C until further use. 

### 2.2. Ovariectomy

Models of ovariectomy-induced osteoporosis have been described previously [30,31]. Sprague Dawley female rats (10–12-week-old) were OVX or subjected to a sham operation. One week after, the rats were administered PS extract (300 mg/kg body weight in phosphate-buffered saline [PBS]), NS extract (300 mg/kg body weight), 1:1 extract mixture (300 and 600 mg/kg body weight), 2:1 extract mixture (300 and 600 mg/kg body weight), or PBS daily for 6 weeks. Vaginal smear and body weight measurement were performed every Monday for 6 weeks during the administration period. Blood, uteri, vaginas, and brains were collected, and uterine weight was measured at the end of the experiment. Blood analysis and histology evaluation were performed as described above. All rats were maintained in separate cages with a constant temperature (23–25 °C) and humidity (45–50%), and a 12-h light/dark cycle. The rats were provided food and water *ad libitum*. 

All animal experiments were approved by the Institutional Animal Care and Use Committees (Protocol No: JSR-2020-03-002) of Jaseng Spine and Joint Research Institute and conformed with the allowed National Research Council Guidelines.

### 2.3. Facial Flushing Verification Test

To verify the efficacy of the extracts on facial flushing, the surface temperatures of the tail of the ovariectomy-induced menopausal rats showing a vascular response similar to that of human skin tissue was measured. The experiment was designed based on a previous study [32]. The facial flushing verification test group was raised in a polycarbonate cage ([W] 26 × [D] 42 × [H] 18 cm). When the housed rats were moved to a cage for measurement ([W] 40 × [D] 80 × [H] 40 cm) and accommodated in groups, stress occurred between individuals owing to environmental changes and pecking-order disputes. This stress raised the tail surface temperature in the OVX rat models, inducing an effect similar to facial flushing in humans. In this experiment, after 30 min of oral extract administration, the rats were moved to a cage for measurement and then photographed using an infrared camera (Flir T650sc; FLIR Systems, Stockholm, Sweden) for 1 h. The images were analyzed using the ResearchIR v4 program by recording the highest temperature after designating a region of interest at a point 3 cm away from the end of the tail hair of the rats. The analysis was performed based on the results measured up to 15 min, as 0 to 3 min was the stabilization time, showing irregular temperature changes and reduced movement of the rats. The temperature decreased after 15 min.

### 2.4. Vaginal Smear

Vaginal epithelial cells of all rats were observed on slides through smear analysis during the 6-week experimental period. Smeared vaginal cells were dried at room temperature (23–25 °C) and stained with methylene blue for 10 min [33]. The cells were observed under a microscope (Eclipse Ti2; Nikon, Tokyo, Japan), and the percentage of cornified cells was calculated according to a previous study [34] as follows:

$$\text{Percentage of cornified cells}(\%)=\frac{\text{Cornified cells}}{\text{Cornified cells}+\text{Nucleated cells}+\text{Leukocytes}}\times 100$$

### 2.5. Histology and Immunohistochemistry

After the experiments, the separated vagina and uterus were fixed with 10% formaldehyde at 4 °C for 48 h and embedded in paraffin. Paraffin blocks were cut into 5 μm sections and stained with hematoxylin and eosin (H&E) or tartrate-resistant acid phosphatase (TRAP) according to a standard protocol [35]. The vaginal epithelial-layer thickness was measured on Eclipse Ti2 (Nikon) using NIS-Elements BR 5.01 software (Nikon Instruments Inc., Seoul, Republic of Korea). Immunostaining was conducted using a standard protocol. Vagina and uterus sections were incubated with a primary antibody (Table 1) overnight at 4 °C. For immunohistochemical staining, a 3,3′-diaminobenzidine peroxidase substrate detection kit (Vector Laboratories, Inc., Newark, CA, USA) was used to detect immunoactivity, and the tissues were stained with hematoxylin by counterstaining. Images were obtained using a microscope (Eclipse Ti2; Nikon).

### 2.6. Blood Biochemical Analysis

Blood serum was collected from the posterior vena cava of rats. The levels of high-density lipoprotein cholesterol (HDL-C), low-density lipoprotein cholesterol (LDL-C), triglyceride, and alkaline phosphatase (ALP) were analyzed using commercial kits (HDL-C, 2650; TG, 1650; and ALP, 3550; FUJIFILM Wako Pure Chemical Corporation, VA, USA) on a dry chemistry analyzer (DRI-CHEM 7000i; Fujifilm, Japan). 

### 2.7. Hypothalamus Analysis

The levels of 5-hydroxytryptamine (5-HT; MBS702675; MyBioSource, San Diego, CA, USA) in the hypothalamus were determined using an enzyme-linked immunoassay (ELISA) kit according to the manufacturer’s instructions.

### 2.8. Quantification and Statistical Analysis

All data are expressed as the mean ± standard deviation (SD) values from at least three independent experiments. Groups were compared using one-way and two-way analysis of variance (ANOVA). Tukey’s multiple comparison test was performed to determine significant differences within the groups. Statistical significance was set at *p*-value < 0.05.

## 3. Results

### 3.1. Complex Extract of PS and NS Enhanced Vaginal Epithelium Thickness in OVX Rat Models

**Figure 1:**
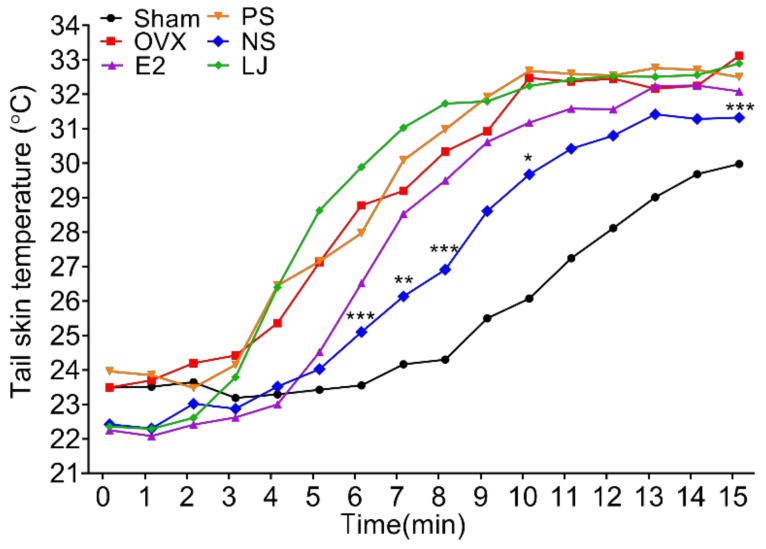
Changes in the tail skin temperature of OVX rats. Data are expressed as the mean ± SD (n = 10). Two-way ANOVA was performed, followed by Tukey’s multiple comparisons test. Asterisks (*) indicate statistical significance (* *p* < 0.05, ** *p* < 0.005, and *** *p* < 0.0005) relative to the OVX group. OVX, ovariectomized; E2, β-Estradiol; PS, *polygonatum sibiricum*; NS, *nelumbinis semen*; LJ, *leonurus japonicus*; SD, standard deviation; ANOVA, analysis of variance.

OVX rats, an animal model of menopause, was used to select complex extract candidates that may enhance the effect of PS on menopause improvement. In addition, changes in the temperature of the tail surface of rats administered NS and LJ were measured for 15 min. The LJ group did not show temperature reduction compared to the OVX group, and PS did not exhibit a significant temperature-reduction effect. However, compared to the OVX group, the NS group exhibited a significantly lower rate of temperature increase, indicating a greater effect than the E2 group (Figure 1). These results supported the use of the complex extract of NS and PS. Therefore, OVX rats were administered PS, NS, or a mixture of both. PS enhanced the size and weight of the uterus, whereas NS did not. Meanwhile, the 2:1 mixture (600 mg/kg) increased the size and weight (Figure S1). 

**Figure 2:**
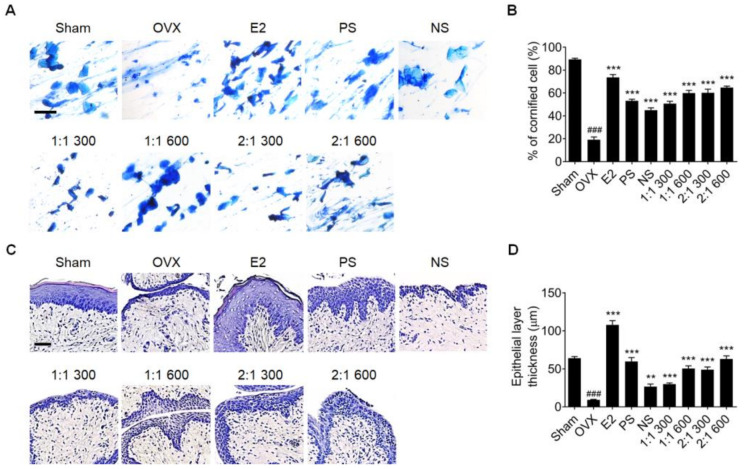
Effects of complex extract of PS and NS on vaginal epithelial tissue. (**A**) Vaginal epithelium cell smears of OVX rats treated with PS, NS, or the mixture for 6 weeks. Cornified cells, nucleated epithelial cells, and leukocytes were observed via methylene blue staining (n = 6). Scale bar = 200 μm. (**B**) Quantitative analyses of the percentage of cornified cells. (**C**) Vaginal tissue slides with H&E staining. (**D**) Quantitative analyses of vaginal epithelial layer thickness. Values are expressed as the mean ± SD. One-way ANOVA was performed, followed by Tukey’s multiple comparisons test. Pound signs (#) indicate statistical significance (### *p* < 0.0005) relative to the sham-operated group. Asterisks (*) indicate statistical significance (** *p* < 0.005, and *** *p* < 0.0005) relative to the OVX group. OVX, ovariectomized; E2, β-Estradiol; PS, *polygonatum sibiricum*; NS, *nelumbinis semen*; H&E, hematoxylin and eosin; SD, standard deviation; ANOVA, analysis of variance.

Vaginal dryness is a representative symptom of the climacteric period in women and occurs when the number of keratinocytes in vaginal epithelial cells decreases and the epithelial cell layer thins [36]. A vaginal epithelial-cell smear was performed to determine the effect of the mixture on the vaginal tissues of OVX rats. Only leukocytes were present in the smears of OVX rats, whereas the number of keratinocytes increased in rats administered PS, NS, or the mixture (Figure 2A,B). The efficacy of the mixture was more concentration-dependent than that of PS or NS alone. In addition, in the H&E staining of vaginal tissues and quantification of epithelial layer thickness, the recovery effect of PS and the mixture was higher than that of NS alone (Figure 2C,D). Moreover, the higher the concentration of the mixture and the higher the ratio of PS, the higher the improvement effect.

### 3.2. Complex Extract of PS and NS Increased ERα and ERβ Expression in Vaginal Epithelium 

**Figure 3:**
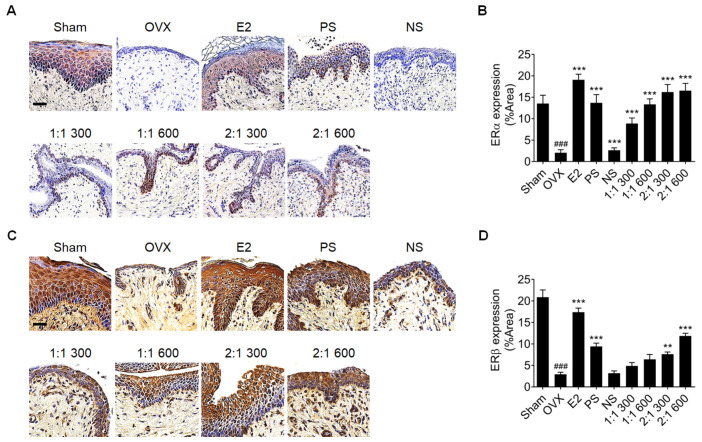
Confirmation of ERα and ERβ expression in vaginal tissues of rats treated with complex extract of PS and NS. (**A**,**C**) Immunostaining of ERα (**A**) and ERβ (**C**) protein expression levels in the vagina of extract-treated OVX and sham-operated rats (n  =  6). Scale bar = 100 μm. (**B**,**D**) Quantitative analyses of protein expression levels of ERα and ERβ, respectively. Values are expressed as the mean ± SD. One-way ANOVA was performed, followed by Tukey’s multiple comparisons test. Pound signs (#) indicate statistical significance (### *p* < 0.0005) relative to the sham-operated group. Asterisks (*) indicate statistical significance (** *p* < 0.005, and *** *p* < 0.0005) relative to the OVX group. OVX, ovariectomized; E2, β-Estradiol; PS, *polygonatum sibiricum*; NS, *nelumbinis semen*; ERα, estrogen receptor alpha; ERβ, estrogen receptor beta; SD, standard deviation; ANOVA, analysis of variance.

Increased vaginal epithelial cell layer increases ERα and ERβ expression [37]. The expression of ERα and ERβ, markers of improved vaginal dryness, were detected based on H&E staining, and ERα levels were found to be higher in all administration groups compared to the OVX group; the higher the ratio and concentration of PS and the more complex the extracts, the greater the effect (Figure 3A,B). However, ERβ expression increased only in the PS and 2:1 mixture groups (Figure 3C,D). These results show that the role of PS is more significant in improving vaginal dryness and that compared with the single extract administration, a complementary effect is observed with the administration of the complex extract with NS.

### 3.3. Complex Extract of PS and NS Ameliorated Depression in OVX Rat Models

Menopause causes anxiety and depression, and studies have shown that postmenopausal women are more likely to develop anxiety disorders than men [3,38]. Serum 5-HT levels in the brain tissue were analyzed to investigate the effect of the PS and NS mixture on improving depression in rats (Table 2). The groups administered PS or NS alone recovered to the level of the sham group as much as the E2 administration group; however, the mixture had a more potent effect than the E2 treatment. 

### 3.4. Complex Extract of PS and NS Improved Blood Lipid Metabolism in OVX Rat Models

**Figure 4:**
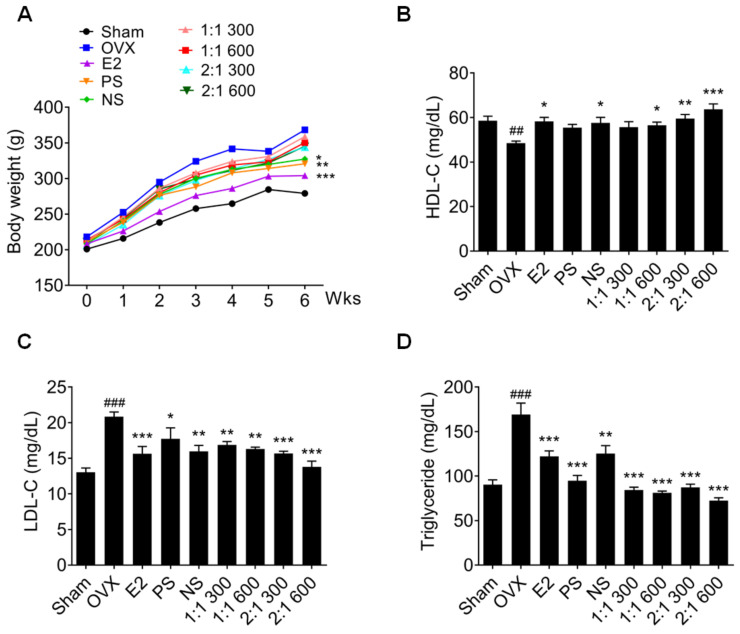
Effects of PS, NS, and mixtures on menopause-related obesity regarding body weight and lipid profile. (**A**) Body weight and (**B**) HDL-C, (**C**) LDL-C, and (**D**) triglyceride levels (n = 6). Values are expressed as the mean ± SD. One-way ANOVA was performed, followed by Tukey’s multiple comparisons test. Pound signs (#) indicate statistical significance (## *p* < 0.005 and ### *p* < 0.0005) relative to the sham-operated group. Asterisks (*) indicate statistical significance (* *p* < 0.05, ** *p* < 0.005, and *** *p* < 0.0005) relative to the OVX group. OVX, ovariectomized; E2, β-Estradiol; PS, *polygonatum sibiricum*; NS, *nelumbinis semen*; HDL-C, high-density lipoprotein; LDL-C, low-density lipoprotein; SD, standard deviation; ANOVA, analysis of variance.

Other menopause symptoms include obesity and low blood-lipid metabolism [3]. The weight of the rats was measured weekly for 6 weeks. Compared to that in the OVX group, the weight of the rats in the PS or NS administration groups was decreased, whereas the weight-loss effect of the complex extract was lower (Figure 4A). However, blood-cholesterol measurement revealed that the recovery effect of the complex extract on blood lipid metabolism was higher than that of the single extract (Figure 4B,D) and that triglyceride was significantly reduced. 

### 3.5. Complex Extract of PS and NS Improved Bone Loss in an OVX Rat Models

**Figure 5:**
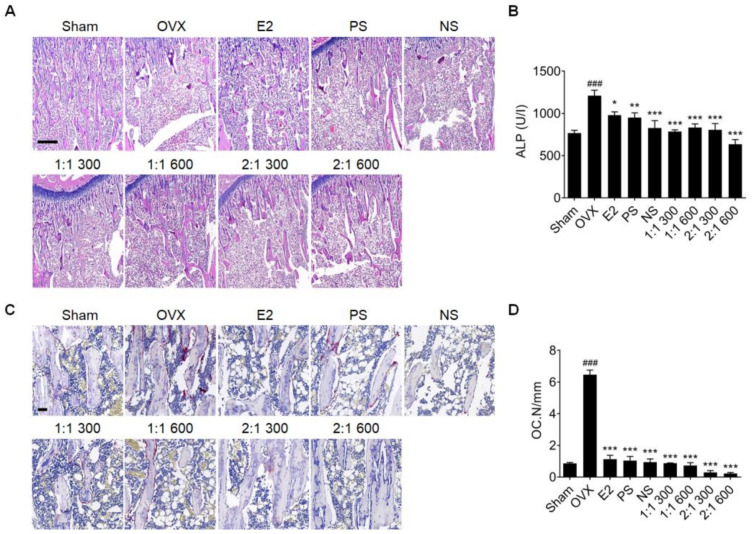
Effect of PS, NS, and mixtures on bone loss in OVX rats. (**A**) Representative H&E staining images of tibial serial sections (n = 6). Scale bar = 500 μm. (**B**) Serum ALP level. (**C**) Representative TRAP staining images of tibial serial sections (n = 6). Scale bar = 50 μm. (**D**) Osteoclast numbers per bone surface (OC.N/mm) are shown. Values are expressed as the mean ± SD. One-way ANOVA was performed, followed by Tukey’s multiple comparisons test. Pound signs (#) indicate statistical significance (### *p* < 0.0005) relative to the sham-operated group. Asterisks (*) indicate statistical significance (* *p* < 0.05, ** *p* < 0.005, and *** *p* < 0.0005) relative to the OVX group. OVX, ovariectomized; E2, β-Estradiol; PS, *polygonatum sibiricum*; NS, *nelumbinis semen*; ALP, alkaline phosphatase; TRAP, tartrate-resistant acid phosphatase; OC, osteoclast; SD, standard deviation; ANOVA, analysis of variance.

To determine the effect of the complex extract of PS and NS in preventing bone loss after menopause, tibia-tissue slides were analyzed using H&E staining. Trabecular bone was formed in all extract-administration groups compared to the OVX group. In the OVX group, trabecular bone was lost, with the complex extract showing the greatest recovery effect (Figure 5A). In addition, based on blood ALP concentrations, compared to the single extract administration group, the complex extract administration group had a greater concentration-dependent inhibitory effect (Figure 5B). The degree of bone loss was analyzed by staining with TRAP, an osteoclast-specific marker. The osteoclast, which was markedly increased in the OVX group, was reduced in all extract administration groups. The reduction effect increased in a concentration-dependent manner in the complex extract administration group compared to that in the PS or NS administration group (Figure 5C,D). 

### 3.6. Complex Extract of PS and NS Regulated ERβ but Not ERα

**Figure 6:**
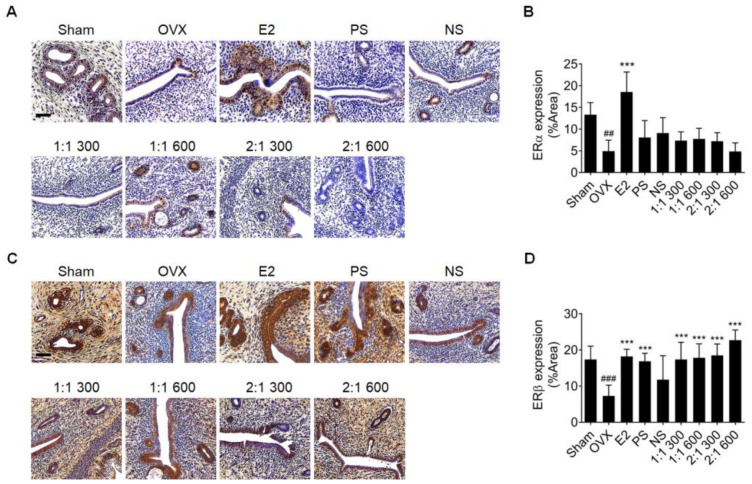
Inhibitory effect of PS, NS, and mixtures on endometrial hyperplasia side effects caused by estradiol. (**A**–**D**) Protein expression confirmation using immunohistochemical analyses of ERα (**A**,**B**) and ERβ (**C**,**D**) in the endometria (n = 6). Scale bar = 50 μm. Values are expressed as the mean ± SD. One-way ANOVA was performed, followed by Tukey’s multiple comparisons test. Pound signs (#) indicate statistical significance (## *p* < 0.005 and ### *p* < 0.0005) relative to the sham-operated group. Asterisks (*) indicate statistical significance (*** *p* < 0.0005) relative to the OVX group. OVX, ovariectomized; E2, β-Estradiol; PS, *polygonatum sibiricum*; NS, *nelumbinis semen*; ERα, estrogen receptor alpha; ERβ, estrogen receptor beta; SD, standard deviation; ANOVA, analysis of variance.

Endometrial hyperplasia due to ERα overexpression was recently identified as a side effect of E2 treatment for menopause [12,13], and PS has been demonstrated to increase ERβ expression without regulating ERα [23]. As the administration of the complex extract of PS and NS is expected to not have side effects, the expression of ERα and ERβ proteins in the uterus of the complex extract administration group was investigated (Figure 6). E2 administration increased ERα and ERβ, whereas PS administration increased only ERβ expression, similar to the previous results. Administration of NS alone did not affect ERα expression but did not also increase ERβ expression. However, when the complex extract was administered, ERα expression gradually decreased, and ERβ expression increased in a concentration-dependent manner compared to those in the single extract administration group, suggesting that the complex extract was superior in terms of the efficacy in improving menopause and the possibility of reducing side effects.

## 4. Discussion

Although various therapies have been developed to alleviate menopause symptoms, existing HRT poses safety concerns owing to its many side effects [6,7]. To date, substances derived from natural plants have been used to treat many diseases, and even with long-term use, few side effects have been reported [39]. Previous studies have investigated the effect of PS on improving menopause symptoms without the risk of side effects, such as endometrial hyperplasia or cancer; however, when used alone in clinical practice, high concentrations of PS are required to promote this improvement. To compensate for these disadvantages, natural products related to menopause or gynecological disease, which show a synergistic effect in improving menopause symptoms, compared to a single administration, and are safe for human use, were screened in a literature review [24,26,40]. 

NS, which was selected as a candidate in this study, is used to treat neurological disorders in women and insomnia [26]. LJ has also been used as a functional food and medicinal herb for menorrhagia, hemorrhage, and uterine function recovery [40,41]. In this study, a simple facial-flushing test was conducted using rats, and NS was selected to form a complex extract with PS and was administered to OVX rat models at various ratios and concentrations to identify its effect on improving menopause symptoms. This study demonstrated that the complex extract was superior to PS or NS alone in improving all menopause symptoms. E2 signaling in the vagina induces epithelium formation by maintaining vaginal epithelium thickness through ERα and ERβ; however, when E2 levels decrease during menopause, ERα and ERβ expression also decreases, resulting in a decrease in the number of keratinocytes and vaginal epithelial thickness, leading to vaginal dryness [42]. In this study, the complex extract of PS and NS restored the vaginal keratinocytes and epithelial thickness by increasing ERα and ERβ expression. The vaginal keratinocyte analysis showed that the administration of the 300 mg/kg mixture had higher efficacy than the single administration of PS and NS, although their contents were 150 + 150 and 200 + 100 mg/kg, respectively, which were less than the 300 mg/kg of a single administration. This finding showed that PS and NS complemented each other and that their mixture increased their efficacy compared to when they were singly administered. In addition, in the H&E staining of vaginal tissue and quantification of epithelial layer thickness, the recovery effect of PS and the mixture was higher than that of NS alone, and the effect increased as the concentration of the mixture and the PS ratio increased. 

Other menopause symptoms include obesity and decreased blood-lipid metabolism [3,43]. The liver uses fatty acids, triglycerides, and cholesterol for metabolism, and E2 helps regulate lipid metabolism in the liver. The decrease in E2 levels owing to menopause results in an increase in LDL-C and triglyceride levels, increasing the risk of cardiovascular disease. In this study, although the weight-loss effect of the complex extract of PS and NS was lower than that of the single extracts, cholesterol measurement revealed that the recovery effect of the complex extract was significantly higher. Triglycerides were significantly reduced; they directly promote cardiovascular diseases and are characterized as being sensitive to the dietary intake, unlike cholesterol [44]. Hence, the triglyceride reduction effect of the complex extract of PS and NS may significantly lower the risk of cardiovascular diseases, such as diabetes and arteriosclerosis, and suppress obesity. 

Additionally, the addition of NS increased the effect of improving menopause compared to the use of PS alone. ERα (ESR1) and ERβ (ESR2) expression levels are essential to evaluate E2 action in endometrial hyperplasia. ERα expression levels have been reported to increase in the endometrium of women with endometrial cancer, resulting in increased estrogenic activity and endometrial proliferation, while ERβ expression remains unchanged [13]. Another study reported an increase in the ERβ/ERα ratio in the endometrium of women with endometrial cancer [45]. PS extract has been reported to relieve menopause symptoms, without endometrial hyperplasia or cancer, through the regulation of the ERβ/ERα ratio, by increasing only ERβ without regulating ERα through the progesterone receptor [23]. In this study, the complex extract with NS was not only more effective in improving menopause symptoms than PS alone but also had no side effects. The complex extract was also found to only increase ERβ without regulating ERα in the endometrium. ERβ plays a major role in facial flushing, obesity, and blood-lipid metabolism [46]. The administration of selective ERβ activators are currently preferred owing to controversy over the increased risk of breast cancer, endometrial cancer, and thromboembolism in the action of ERα [15,16,41,42,43,44,45,46,47,48,49]. However, it is unclear whether these complex extract effects result from direct hormone regulation. To evaluate the safety of the complex extract, the concentration of sex hormones in the blood serum was measured (Figure S2). The complex extract of PS and NS did not change the 17β-estradiol and progesterone levels in the blood serum. Thus, the complex extract of PS and NS demonstrates the feasibility of developing a new menopause therapy that has few side effects and is safe with no risk of direct hormone regulation. 

## 5. Conclusions

The results of this study suggest that the complex extract of PS and NS increased the menopause-symptom improvement effect through complementary action compared to the use of PS alone; it alleviated menopause symptoms without risk of endometrial cancer and hypertrophy, by increasing ERβ alone without regulating ERα in the endometrium. These findings suggest that the complex extract of PS and NS may be a new treatment for menopause with fewer side effects than conventional synthetic hormone administration treatments, such as HRT. 

## Figures and Tables

**Table 1:** Antibodies used in the immunohistochemical assay.

Antibody	Company	Dilution	Product no.
ERα	Abcam	1:50	ab32063
ERβ	Invitrogen	1:100	PA1-310B

ERα, estrogen receptor alpha; ERβ, estrogen receptor beta.

**Table 2:** Serotonin concentrations in brain tissue.

Variables	SHAM	OVX	E2	PS	NS	1:1300	1:1600	2:1300	2:1600
5-HT (pg/mL)	25.25 ± 2.23	18.94 ^#^ ± 0.49	24.08 ± 1.41	24.22 ± 0.89	26.02 ** ± 1.54	25.22 *± 0.62	30.73 *** ± 3.83	31.19 ***± 2.35	36.48 *** ± 3.70

5-HT levels from the hypothalamus of OVX rats were measured using ELISA. Data are expressed as the mean ± SD (n = 6). One-way ANOVA was performed, followed by Tukey’s multiple comparisons test. Pound signs (#) indicate statistical significance (# *p* < 0.05) relative to the sham-operated group. Asterisks (*) indicate statistical significance (* *p* < 0.05, ** *p* < 0.005, and *** *p* < 0.0005) relative to the OVX group. OVX, ovariectomized; E2, β-Estradiol; PS, *polygonatum sibiricum*; NS, *nelumbinis semen*; 5-HT, 5-hydroxytryptamine; ELISA, enzyme-linked immunosorbent assay; SD, standard deviation; ANOVA, analysis of variance.

## Data Availability

The data presented in this study are available upon request from the corresponding author.

## Supplementary Materials

The following supporting information can be downloaded at: https://www.mdpi.com/article/10.3390/nu15112443/s1, Figure S1: Effects of PS, NS, and mixtures on uterine weight; Figure S2: Effect of PS, NS, and mixtures on E2 and progesterone levels in the serum of OVX mice.
