# Supplementary material for: Complex Extract of Polygonatum sibiricum and Nelumbinis semen Improves Menopause Symptoms via Regulation of Estrogen Receptor Beta in an Ovariectomized Rat Model
Source: Nutrients. 2023 May 24;15(11):2443. doi: 10.3390/nu15112443 (PMC10255448; doi:10.3390/nu15112443)
Supplement: Supplementary file 1 [file nutrients-15-02443-s001.zip › nutrients-2373306-supplementary.pdf]

Supplementary Information

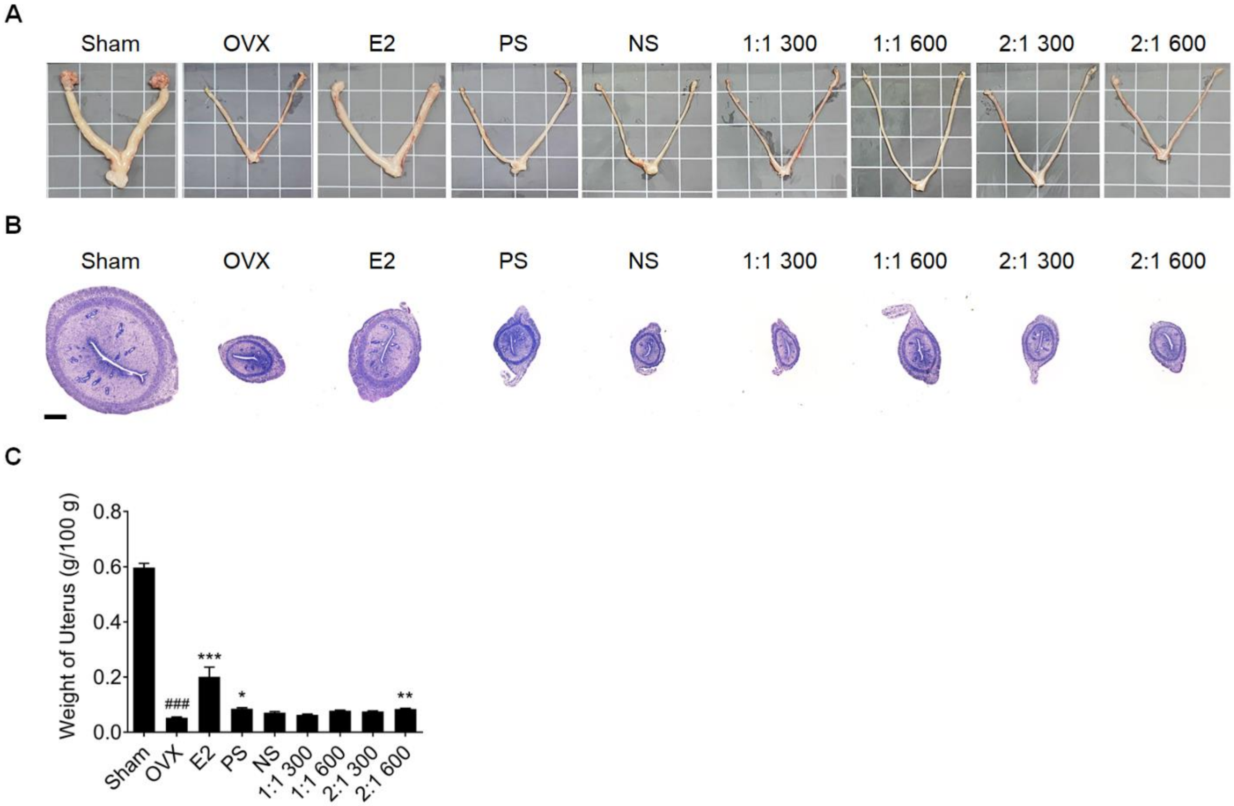

Figure S1. Effects of PS, NS, and mixtures on uterine weight. (**A**) Photograph of the uterus. (**B**) Uterus sections from rats treated with estradiol, PS, NS, or mixtures stained with H&E (n = 6). Scale bar = 100 μm. (**C**) Uterus weight was measured at the end of the 6-weeks treatment period. Values are expressed as the mean ± SD. One-way ANOVA was performed, followed by Tukey's multiple comparisons test. Pound keys (#) indicate statistical significance (### $p < 0.0005$) relative to the sham-operated group. Asterisks (*) indicate statistical significance (* $p < 0.05$, ** $p < 0.005$, and *** $p < 0.0005$) relative to the OVX group. OVX, ovariectomized; E2, β-Estradiol; PS, *polygonatum sibiricum*; NS, *nelumbinis semen*; H&E, hematoxylin and eosin; SD, standard deviation; ANOVA, analysis of variance.

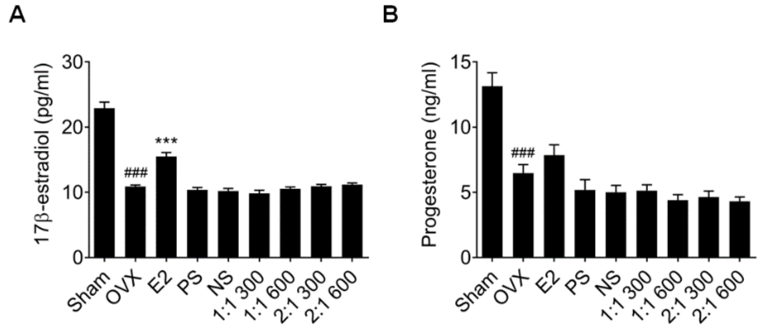

Figure S2. Effect of PS, NS, and mixtures on E2 and progesterone levels in the serum of OVX mice. Serum levels of (**A**) E2 and (**B**) P4 from OVX mice were measured using ELISA (n = 6). Values are expressed as mean ± SEM. One-way ANOVA was performed, followed by Tukey's multiple comparisons test. Pound keys (#) indicate statistical significance (### $p < 0.005$) relative to the sham-operated group. Asterisks (*) indicate statistical significance (*** $p < 0.005$) relative to the OVX group. OVX, ovariectomized; E2, β-Estradiol; PS, *polygonatum sibiricum*; NS, *nelumbinis semen*; ELISA, enzyme-linked immunosorbent assay; SD, standard deviation; ANOVA, analysis of variance.
